# Editorial for Special Issue “Molecular Insights into Food-Derived Natural Products and Their Biological Activities—2nd Edition”

**DOI:** 10.3390/cimb48101063

**Published:** 2026-10-15

**Authors:** Yanfang Li

**Affiliations:** Department of Chemistry and Biochemistry, Ohio University, Athens, OH 45701, USA; yfl0820@ohio.edu

Food-derived natural products contain a broad range of bioactive constituents, including polyphenols, flavonoids, saponins, terpenoids, alkaloids, peptides, and other secondary metabolites. Recent studies have increasingly focused on how these compounds interact with specific molecular targets and influence signaling, inflammatory, and metabolic pathways [1,2]. At the same time, bioavailability, metabolism, food-matrix effects, and gut microbial transformation can substantially influence the biological activity of these compounds *in vivo* [3,4]. Advances in metabolomics and computational approaches are also creating new opportunities to connect the complex chemical composition of foods with experimentally testable biological functions [5].

The second edition of the Special Issue “Molecular Insights into Food-Derived Natural Products and Their Biological Activities” includes eight contributions, comprising five original research articles [6,7,8,9,10] and three reviews [11,12,13]. Together, these studies address oxidative and inflammatory responses, molecular targets and signaling pathways, dose-dependent effects, bioconversion, and factors affecting the biological relevance of food-derived bioactives.

Simeonova et al. investigated the nephroprotective effects of *Tanacetum balsamita* leaf extract in a rat model of metabolically induced renal injury [6]. The extract improved several biochemical and urinary indicators of renal injury and enhanced antioxidant defense; the authors suggested that these effects could be associated with its caffeoylquinic acids and methoxylated flavones and flavonols [6]. Gregorio et al. examined the soy isoflavones daidzein and genistein in 3T3-L1 adipocytes and reported biphasic, concentration-dependent regulation of *Slc2a4* gene expression and GLUT4 protein expression, mediated primarily through estrogen receptor 1 (ESR1) [7]. Low concentrations increased *Slc2a4* mRNA and GLUT4 protein expression, whereas high concentrations reduced them. These results also illustrate the importance of concentration when interpreting the biological effects of food-derived bioactives.

Shin et al. evaluated a bioconverted blueberry extract in human dermal microvascular endothelial cells exposed to oxidative stress [8]. The extract reduced reactive oxygen species accumulation, preserved mitochondrial membrane potential, improved endothelial migration and tube formation, and attenuated excessive MAPK activation. This study also highlights bioconversion as a potential approach for modifying the biological activity of plant-derived ingredients. Complementing these cell-based findings, Tabolacci et al. provided a human population perspective through a longitudinal analysis of chili pepper consumption and circulating inflammatory biomarkers in the Moli-sani Study [9]. Increased chili pepper consumption over approximately 12.7 years was associated with lower circulating TNF-α concentrations, although associations were not consistent across all inflammatory markers. The study provides useful human observational evidence alongside the mechanistic and preclinical work represented in this collection of papers.

Kafadar and Yıldızhan investigated oxidative stress, inflammation, and ferroptosis-related alterations in cecal ligation and puncture-induced colonic injury using ferrostatin-1 [10]. Treatment was associated with improvements in oxidative and inflammatory parameters and changes in ACSL4 and GPX4 immunoreactivity. Although ferrostatin-1 is a synthetic ferroptosis inhibitor rather than a food-derived natural product, this study broadens the mechanistic scope of the Special Issue by examining oxidative stress, inflammation, lipid peroxidation, and ferroptosis-related alterations in intestinal injury.

The three reviews address additional molecular and therapeutic aspects of natural products. Moon reviewed natural products, particularly saponins, as potential allosteric inhibitors of SHP2, a key regulator of RAS/MAPK and PI3K/AKT signaling [11]. The review discusses the potential of saponin-based compounds to target the SHP2 allosteric tunnel site and considers structural optimization and combination strategies for future development. Harej Hrkać et al. examined herbs and spices associated with the Mediterranean diet as potential protectants against blue-light-induced retinal degeneration [12]. Their review highlights compounds including rosmarinic acid, carnosic acid, eucalyptol, eugenol, and thymol in relation to oxidative stress and inflammation. Jaiswal and Lee reviewed spinacetin, a flavonol originally isolated from spinach, and summarized its reported antioxidant, anti-inflammatory, anticancer, antidiabetic, and organ-protective activities together with molecular targets and pathways such as COX-2, SIRT1/AMPK/mTOR, MAPK, NF-κB, and AKT signaling [13]. They also noted that much of the current evidence remains preclinical and that pharmacokinetic and safety information is still limited.

Several themes emerge from the studies in this Special Issue. Mechanistic characterization is becoming increasingly important, with greater attention to specific targets and signaling pathways rather than general descriptions of antioxidant or anti-inflammatory activity. The studies also demonstrate the importance of concentration, metabolism, processing, bioconversion, and experimental models when evaluating natural products. Recent studies of polyphenol bioavailability and gut microbial metabolism further show that the molecular species reaching target tissues may differ from those originally present in foods [3,4]. Meanwhile, the identification of molecular targets and the integration of metabolomics with computational prioritization can also help connect complex food chemical profiles with testable mechanistic hypotheses [2,5].

Future studies should give greater attention to physiologically relevant concentrations, food-matrix effects, metabolite exposure, interindividual variation, and the standardization of natural-product preparations. Mechanistic findings from *in vitro* systems should be validated in appropriate *in vivo* models and, where possible, in well-designed human studies. Combining analytical chemistry and metabolomics with molecular, computational, and nutritional approaches should help determine which mechanistic findings are most likely to remain relevant under realistic exposure conditions.

Overall, the studies presented in this Special Issue demonstrate the chemical and biological diversity of natural products and provide new insights into the molecular mechanisms relevant to metabolism, oxidative stress, inflammation, vascular and renal function, and ocular health. We hope that this second edition will stimulate further interdisciplinary research linking food composition and natural-product chemistry with molecular mechanisms and physiologically relevant biological outcomes.
